# Phylogenetic analysis, morphological studies, element profiling, and muscarine detection reveal a new toxic *Inosperma* (Inocybaceae, Agaricales) species from tropical China

**DOI:** 10.3389/fmicb.2023.1326253

**Published:** 2023-12-07

**Authors:** Yu-Ling Zhou, Lun-Sha Deng, Shu-Dong Yang, Chao-Feng Liu, Yu-Guang Fan, Wen-Jie Yu

**Affiliations:** ^1^Hainan Institute for Food Control, Key Laboratory of Tropical Fruits and Vegetables Quality and Safety for State Market Regulation, Haikou, China; ^2^Key Laboratory of Tropical Translational Medicine of Ministry of Education, Hainan Key Laboratory for R & D of Tropical Herbs, Tropical Environment and Health Laboratory, School of Pharmacy & International School of Public Health and One Health, Hainan Medical University, Haikou, Hainan, China; ^3^Changchun Food and Drug Inspection Center, Changchun, Jilin, China

**Keywords:** new species, tropical China, Old World tropical clade 2, muscarine, element

## Abstract

Tropical Asian collections of *Inosperma* are usually poisonous mushrooms that have caused many poisoning incidents. However, the species diversity and the toxic mechanisms of these *Inosperma* species are still unclear. In this study, we describe the discovery of *Inosperma wuzhishanense* sp. nov. from Wuzhishan City, Hainan Province, tropical China. The new species was identified based on morphological and multi-locus (ITS, nrLSU, and *RPB2*) phylogenetic analyses. The new species is characterized by its reddish-brown pileus, fibrillose stipes with finely protruding fibrils, rather crowded lamellae, smooth and ellipsoid basidiospores, and mostly clavate, thin-walled cheilocystidia. The new species is phylogenetically nested in the Old World tropical clade 2 and is sister to the tropical Indian taxa *I. akirnum*. Detailed descriptions, color photos of the new species, and comparisons with its closely related species are provided. Additionally, the muscarine content of the new species was analyzed by ultra-performance liquid chromatography/tandem mass spectrometry (UPLC–MS/MS). The muscarine contents ranged from 4,359.79 ± 83.87 mg/kg to 7,114.03 ± 76.55 mg/kg, 2,748.37 ± 106.85 mg/kg to 4,491.35 ± 467.21 mg/kg, and 2,301.36 ± 83.52 mg/kg to 2,775.90 ± 205.624 mg/kg in the stipe, pileus, and lamellae, respectively. The elemental composition and concentration were determined using inductively coupled plasma-mass spectrometry (ICP-MS). A total of 24 elements were detected. Among the heavy metals detected, arsenic showed the highest level of toxicity with a concentration of 36.76 ± 0.43 mg/kg.

## 1 Introduction

The genus *Inosperma* (Kühner) Matheny and Esteve-Rav. was first established as *Inocybe* sect. *Inosperma,* with *Inocybe calamistrata* Kühner as its type species in the past (Kühner, 1980). Recently, however, Matheny et al. (2020) elevated it as a genus of the Inocybaceae family with approximately 55 recognized species. Now, the number of *Inosperma* spp. listed in the Index Fungorum database has rapidly increased to 86 (as of 1 August 2023) with seven newly discovered species from Africa (Aïgnon et al., 2021, 2023; Buyck et al., 2021), five from Europe (Cervini et al., 2021; Bandini et al., 2021, 2022), and nine from China (Deng et al., 2021a,b, 2022a; Li S. N. et al., 2022). There are six major phylogenetic clades in the genus, *viz.*, the *I. africanum* lineage, sect. *Cervicolores*, *I. misakaense* lineage, Maculatum clade, Old World tropical clade 2, and Old World tropical clade 1 (Deng et al., 2021a,b, 2022a). All species in the Old World tropical clade 2 are mainly distributed in tropical Asia and the adjacent regions, including India, Thailand, Papua New Guinea, and tropical China (Vrinda et al., 1996; Latha and Manimohan, 2017; Deng et al., 2021a,b, 2022a). They often exhibit yellowish brown basidiomata, silky smooth, fibrillose-rimulose to rimose pileus, nearly smooth stipe, crowded lamellae, smooth and ellipsoid basidiospores, and thin-walled cheilocystidia. In the field, they usually grow gregariously as large, discrete clusters under Fagaceae, Dipterocarpaceae, or Pinaceae trees. A global ITS-phylogeny using sequence data from GenBank revealed that there are still many undescribed *Inosperma* taxa from different regions of the world (Deng et al., 2022b).

Poisoning by muscarine-containing mushrooms causes a rapid onset (15–120 min) of classic symptoms of parasympathetic stimulation. The main symptoms are increased sweating, salivation, and lachrymation, and other symptoms include pupil constriction, blurred vision, urgent or painful micturition, nasal discharge or congestion, asthma, bronchoconstriction, hypotension, bradycardia, skin flushing, watery diarrhea, vomiting, abdominal pain, and colic. The more rapid the onset, the more severe the intoxication, and severe poisoning can be lethal, although death is a rare outcome and unlikely to occur with appropriate atropine treatment (White et al., 2019). It has been reported that *Inosperma* spp. contain muscarine, and certain species are known to have caused many mushroom poisoning incidents (Chandrasekharan et al., 2020; Li W. W. et al., 2021; Parnmen et al., 2021; Deng et al., 2022a; Li H. J. et al., 2023). However, little is known about the muscarine content in most *Inosperma* species, and only nine species have been confirmed to be muscarine-positive so far. The literature suggests that *Inosperma* spp. from Old World tropical clade 2 usually have high concentrations of muscarine (Sailatha et al., 2014; Chandrasekharan et al., 2020; Latha et al., 2020; Parnmen et al., 2021; Deng et al., 2021a, 2022a; Li H. J. et al., 2022, 2023). In addition, studies have shown that long-term exposure to toxic elements through mushroom consumption may be hazardous to human health (Fu et al., 2020; Nowakowski et al., 2021). However, little is known about the types and concentrations of toxic elements in members of the *Inosperma* genus (Bizio et al., 2020).

Here, we report a new *Inosperma* species from Wuzhishan City, in the tropical region of China. This species shares some features with species in Old World tropical clade 2. Comprehensive macro- and microscopic descriptions, color photographs, and illustrations of the new species are provided in this study. In addition, the contents of muscarine and other elements were determined to verify the uniqueness of the new species and to evaluate its potential toxicity.

## 2 Materials and methods

### 2.1 Morphological and phylogenetic studies

#### 2.1.1 Sample collections and morphological studies

Fresh basidiomata were photographed, noted, and collected in the field; the color codes of descriptions were derived from Kornerup and Wanscher (1978). An electronic drier was used to dry the collected specimens at 45°C overnight. Microscopic features were observed in these dried specimens rehydrated in KOH solution (5%) or stained with Congo red solution (1%) if needed. The methods of measuring followed Ge et al. (2021) with small modifications. At least 20 basidiospores were randomly selected for each individual specimen, and the apiculus was excluded when measured. The abbreviation [a/b/c] represented “a” basidiospores measured from “b” basidiomata of “c” specimens. The size of basidiospores “length × width” was indicated as (d) e–f–g (h) × (i) j–k–l (m), where “d” is the minimum length, “e–g” represents 5 to 95% values, “h” is the maximum value of length, and the bolded “f” means the average value. Alphabets in width “i,” “j–l,” “m,” and “k” are shown with the same meaning. Q refers to the ratio of length/width of basidiospore in side view, and Q_m_ ± SD is the average value of Q ± the standard deviation of all Q values. Total dried specimens were deposited in the Herbarium of Changbai Mountain Nature Reserve (ANTU) with FCAS numbers.

#### 2.1.2 DNA extraction, PCR amplification, and sequencing

Genomic DNA was extracted from dried materials using a NuClean Plant Genomic Kit (ComWin Biotech, Beijing). Three genes (ITS, nrLSU, and *RPB2*) were amplified and sequenced with the primers: ITS1F/ITS4 for ITS (Gardes and Bruns, 1993), LR0R/LR7 for nrLSU (Vilgalys and Hester, 1990), and bRPB2-6F/bRPB2-7.1R for *RPB2* (Matheny, 2005). The total volume of polymerase chain reaction (PCR) mixture solution was 25 μL, including 9.5 μL of dd H_2_O, 12.5 μL of 2 × Taq Plus Master Mix (Dye), 1 μL of each primer, and 1 μL of template DNA. The PCR conditions for ITS, nrLSU, and *RPB2* were initial 95°C for 1 min, followed by 35 cycles at 95°C for 30 s, annealing at 52°C for 1 min, elongation at 72°C for 1 min, and a final extension at 72°C for 8 min (Yan J. Q. et al., 2022). The amplified PCR products were sent to Sangon Biotech (Shanghai) Co., Ltd. for purification and sequencing.

#### 2.1.3 Molecular phylogenetic analyses

Newly generated sequences in this study were verified and submitted to GenBank,^1^ and related sequences from recent literature were downloaded from GenBank and literature (Latha and Manimohan, 2017; Aïgnon et al., 2021, 2023; Cervini et al., 2021; Parnmen et al., 2021; Deng et al., 2021a,b, 2022a), and are listed in Supplementary Table S1. Individual genes were aligned using the online service of MAFFT version 7, with E-INS-i iterative refinement method,^2^ respectively (Katoh et al., 2019). BioEdit version 7.0.9.0 (Hall, 1999) was used to manually improve the aligned sequences when necessary. MrModeltest v.2.3 was performed for each locus to select the best-fit evolutionary model with the Akaike Information Criterion (AIC) (Nylander et al., 2008). Three genes were concatenated into single combined alignments (*RPB2*-ITS-LSU) using MEGA v.5.02 (Tamura et al., 2011). The maximum likelihood (ML) analyses were conducted with the IQ-TREE web server^3^ with 1,000 ultrafast bootstrap replicates (Trifinopoulos et al., 2016). The Bayesian tree (BI) was generated using MrBayes v.3.2.7a. Four Markov chains (MCMCs) were set to run for 100 million generations and automatically terminated using the stoprul and stopval commands when the standard deviation of the split frequencies fell below 0.01, with sampling for every 100 generations. The first 25% of trees were in the burn-in phase and were discarded (Ronquist et al., 2012). The resulting trees were viewed through FigTree version 1.4.3. (Rambaut, 2009). The final alignment was registered on TreeBASE (ID: 30769).

### 2.2 Muscarine detection

#### 2.2.1 Sample preparation

The dried mushroom materials were divided into stipe, pileus (without the lamellae), and lamellae and finely ground into homogenized powder, respectively. Approximately 10–20 mg dry weight was extracted with 2 mL of methanol–water (5:95, *V/V*). The mixture was vortexed for 30 min at first, then ultrasound-assisted extraction for another 30 min (10°C, 33 Hz). The mixture was centrifuged at 10,000 rpm for 5 min, and the supernatant was collected. A 0.22 μm organic filter membrane was used to filtrate the supernatant before UPLC–MS/MS analysis, and acetonitrile-water (7:3, *V/V*) was applied to a diluted solution if needed. *Lentinula edodes* (Berk.) Pegler was treated as the blank sample. The analytical results are reported as mean ± SD mg/kg, where the mean is the average content of muscarine and SD represents the standard deviation.

#### 2.2.2 UPLC-MS/MS analysis

The detailed parameter settings of UPLC-MS/MS followed Xu et al. (2020) and Deng et al. (2022a) with some modifications. A Waters ACQUITY UPLC I-Class Plus/Xevo TQ-XS system with an ACQUITY UPLC BEH Amide (2.1 mm × 100 mm, 1.7 μm) column was applied to the UPCL-MS/MS analysis. The gradient elution was used at a flow rate of 0.3 mL/min as follows (mobile phase A, 0.05% formic acid aqueous solution; mobile phase B, acetonitrile): 0.0–0.1 min 90.0% A, 10.0% B; 0.1–1.5 min 10.0% A, 90.0% B; 1.5–2.0 min 10.0% A, 90.0% B; and 2.0–2.1 min 90.0% A, 10.0% B; 2.1–3.0 min 90.0% A, 10.0% B. For UPLC separations, the temperature of the chromatographic column was set at 40°C. The injection volume was 2.0 μL. The [M + H] ^+^ ion of muscarine (m/z 174.200) was selected as the parent ion, and ions of 57.000 as well as 97.000 were used for qualitative and quantitative detection, respectively. The rest of the MS parameters were set the same of Xu et al. (2020).

### 2.3 Common and trace elemental analysis

#### 2.3.1 Sample preparation

All experimental samples were carefully cleaned and separated from soil residue. The dried and powdered sample (0.25 g) was digested completely using 7 mL of HNO_3_ (67–70%) microwave and it was heated at 110°C on a graphite digestion apparatus for 30 min. After cooling, digested samples were diluted with purified water to a volume of 50 mL.

#### 2.3.2 Chemicals and instruments

All digested reagents were trace metal grade and purchased from Fisher Chemical. The ICP-MS was used for the determination of elements (In, Bi, and Ge are internal standard). The conditions of the ICP-MS are as follows: radio frequency power: 1.60 kw; carrier gas: 99.999% Ar; plasma gas flow rate: 15.0 L/min; auxiliary gas flow rate: 1.2 L/min; nebulizer gas flow rate: 1.02 L/min; detector mode: standard mode; integration time: 1000 ms. The analysis process, triplicate samples, blanks, precision, and rates of recovery were referred to GB5009. 268–2016 Chinese National Standard for Food Safety-Determination of Multiple Elements in Food.

## 3 Results

### 3.1 Phylogenetic analyses

**Figure 1:**
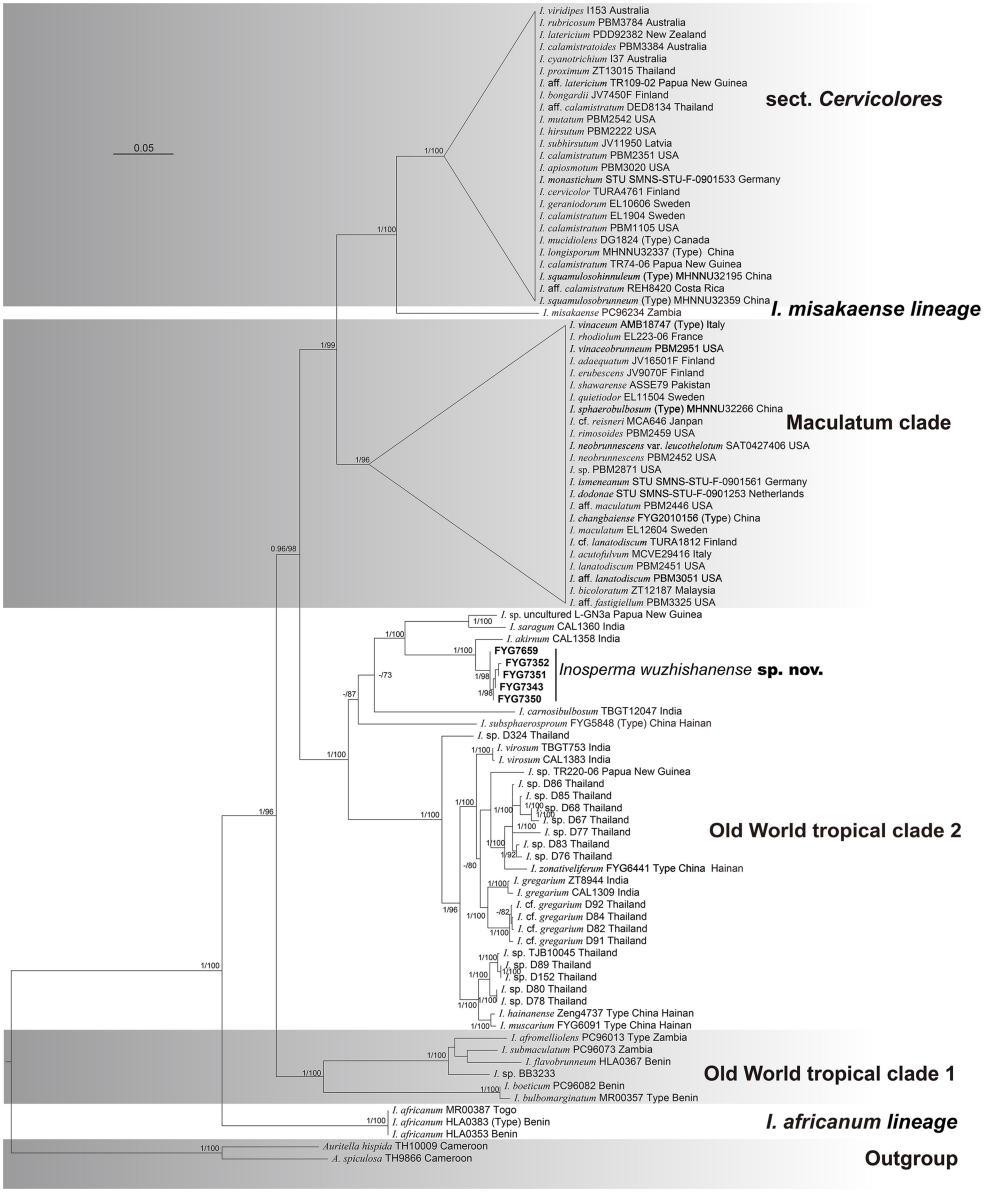
Phylogram generated by Bayesian (BI) analyses based on sequences of a combined dataset from nuclear genes, rooted with *Auritella hispida* and *A. spiculosa*. Bayesian inference (BI-PP) ≥ 0.95 and ML bootstrap proportions (ML-BPs) ≥ 70 are represented as BI-PP/ML-BP. *Inosperma wuzhishanense* is the newly described taxa; Scale bar = 0.05.

The final multi-locus concatenated dataset (*RPB2*-ITS-LSU), which included 96 taxa, had 3,200 nucleotide sites, comprising 860 bp ITS, 1,560 bp LSU, and 780 bp *RPB2*, of which 1,781 bp were constant sites and 1,004 bp were parsimony informative sites. All gene regions resulted in the GTR + I + G model. Only the phylogenetic tree inferred from the maximum likelihood (ML) strategy is provided because of the topological consistency between ML and BI. The values of maximum likelihood bootstrap (MLB) as well as Bayesian posterior probabilities (BPPs) are presented in Figure 1. A total of 15 newly generated sequences (5 ITS, 5 LSU, and 5 *RPB2*) have been submitted to GenBank. *Auritella hispida* Matheny and T.W. Henkel and *A. spiculosa* Matheny and T.W. Henkel were treated as outgroups. All of our collections of the novel species were nested in Old World tropical clade 2 and were grouped into an independent lineage with strong support (MLB = 100%, BPP = 1). The phylogenetic analysis shows that the new species is sister to *I. akirnum* (K.P.D. Latha and Manim.) Matheny and Esteve-Rav. (MLB = 100%, BPP = 1), and the distinct lineage that formed by these two taxa is close to *I. saragum* (K.P.D. Latha and Manim.) Matheny and Esteve-Rav. (MLB = 100%, BPP = 1) and an undescribed species from Papua New Guinea (*Inosperma* sp. L-GN3a) (MLB = 100%, BPP = 1).

### 3.2 Taxonomy

**Figure 2:**
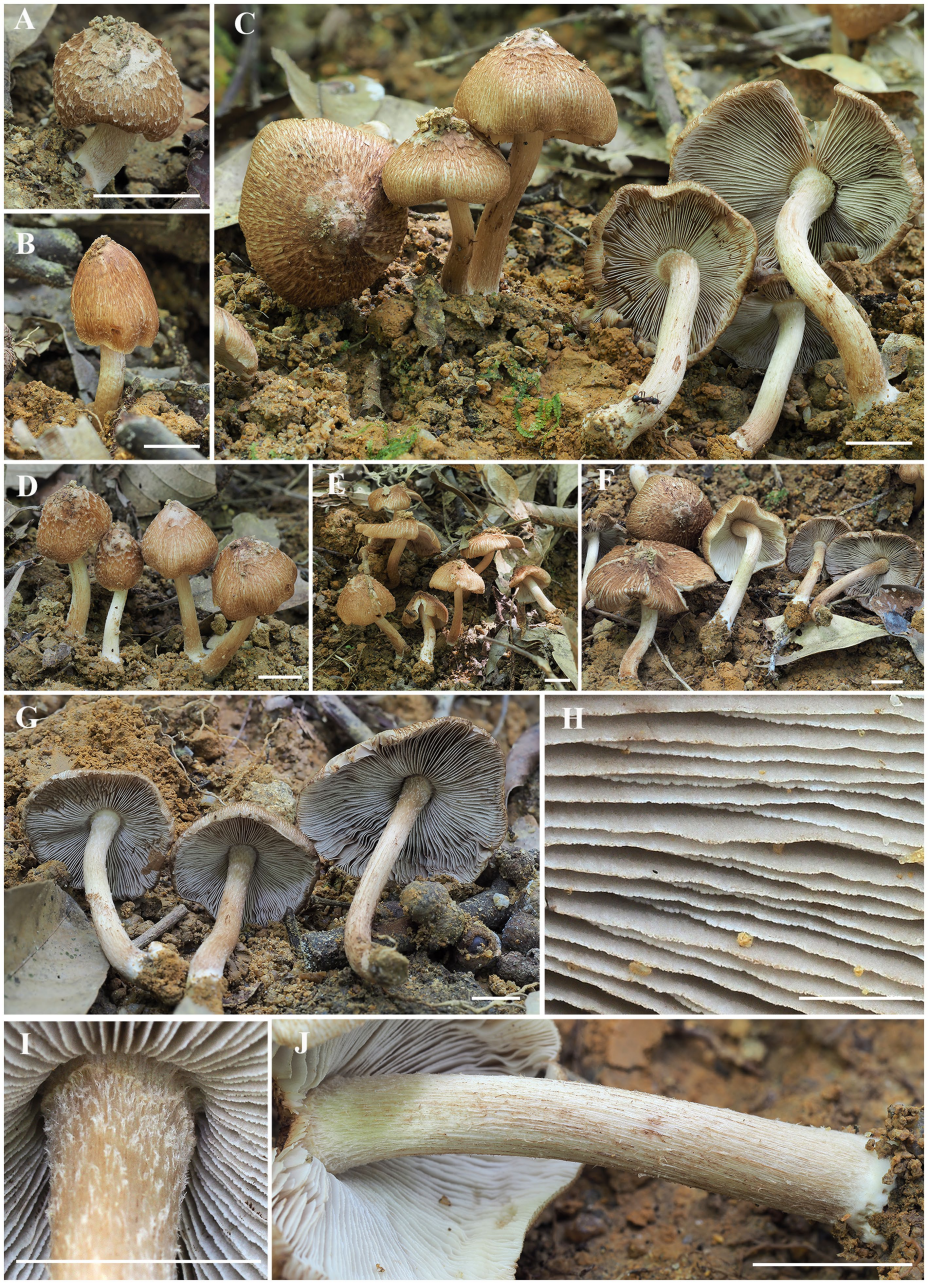
Macroscopic characteristics of *I. wuzhishanense*. **(A–F)** Basidiomata; **(G)** lamellae; **(H)** lamellae edge; and **(I–J)** stipe surface. Scale bars: A–J = 10 mm. Photos by Y.-G. Fan.

**Figure 3:**
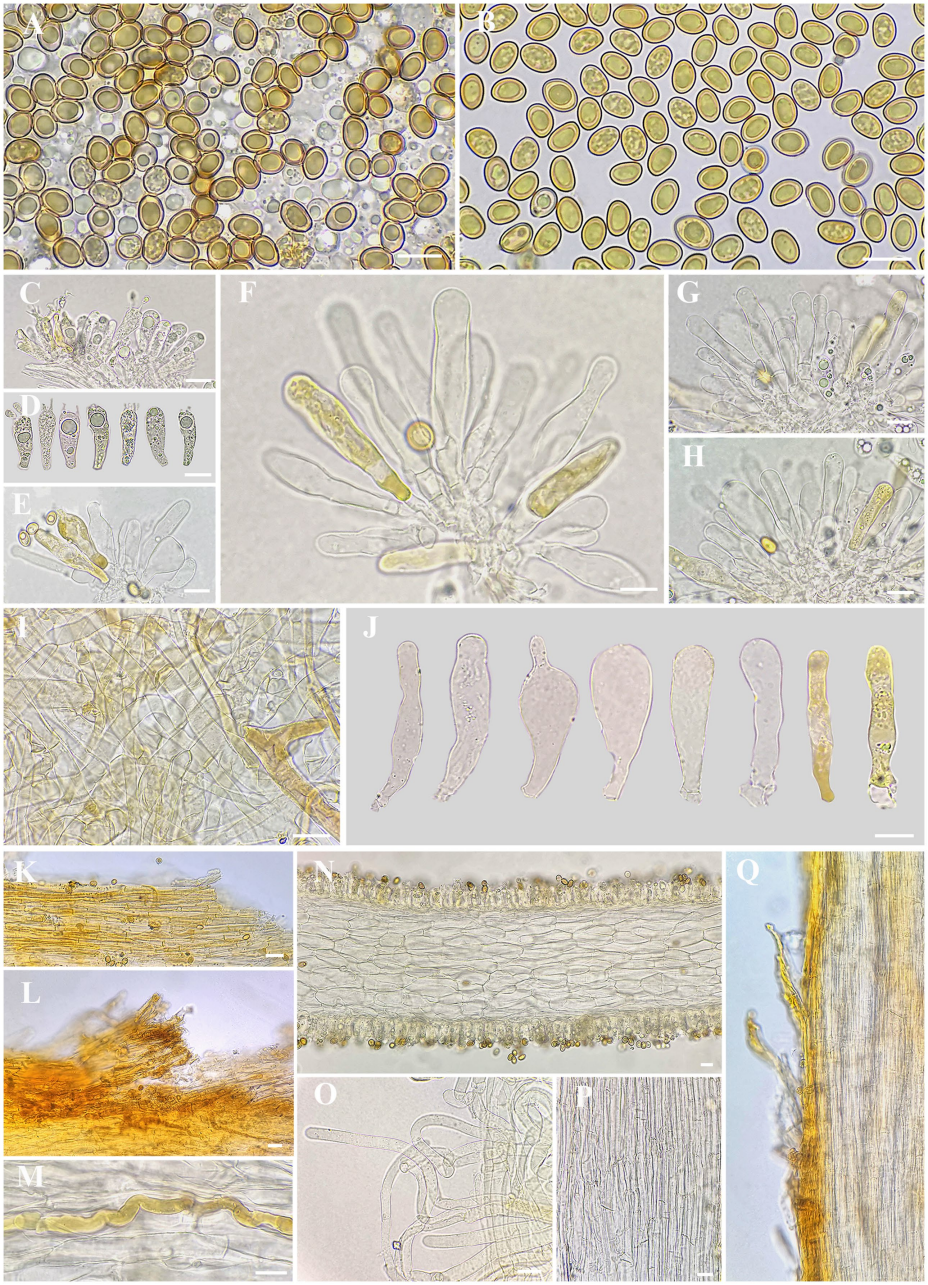
Microscopic features of *I. wuzhishanens*e. **(A,B)** Basidiospores; **(C,D)** basidia; **(E–H,J)** cheilocystidia in clusters; **(I)** veilpellis; **(K–L)** pileipellis; **(M)** oleiferous hyphae; **(N)** cross-section of lamella; **(O)** extended terminal inflated hyphae in upper stipe surface; **(P)** stipe trama; and **(Q)** stipitipellis. Scale bars: A–Q = 10 μm. Photos by L.-S. Deng.

*Inosperma wuzhishanense* sp. nov. Y.G. Fan, L.S. Deng, and W.J. Yu (Figures 2, 3).

*MycoBank*: 850631.

*Etymology*: “*wuzhishanense*” means Wuzhi Mountain, where the type specimen was collected.

*Holotype*: China, Hainan Province, Wuzhishan City, Wuzhishan Nature Reserve, July 29, 2022, Y.G. Fan, W.J. Yu, and L.S. Deng, FYG7659 (FCAS3801).

*Diagnosis*: The new species is characterized by its scaly reddish-brown pileus, fibrillose stipes with finely protruding fibrils, rather crowded lamellae, smooth and ellipsoid basidiospores measuring 8.0–9.2 × 5.0–6.2 μm, and mostly clavate, thin-walled cheilocystidia >50 μm in length. It occurs under fagaceous trees in tropical China.

*Descriptions*: Basidiomata small to medium-sized. Pileus 20–46 mm in diam., conical when very young, becoming convex to plano-convex when mature, with an indistinct umbo in young and middle age, becoming obtusely umbonate with age, margin incurved when very young, still incurved to slightly decurved for a long time upon maturity; surface dry, smooth and covered with appressed to slightly protruding patches of whitish (4A1) veils at first, then veil remnants disappeared soon and only present at the center with age; becoming fibrillose-rimulose, appressed fibrillose to rimose, radially covered with slightly recurved to appressed scales from the center to margin; dark reddish-brown (5B4) around the center, yellowish brown (5A2) to reddish-brown (5A3) elsewhere, background ivory whitish (3A1) to slightly grayish white (3B1). Lamellae 2–3 mm wide, adnexed, rather crowded, whitish (3A1) at first, then dirty white (3B1) to grayish white (3C1) when matured, yellowish brown (3B2) after drying, alternatively distributed with at least three unequal lamella, edge fimbriate, slightly serrate to wavy. Stipe 35–65 × 4–5 (−6) mm, central, solid, terete, slightly swollen at apex, base entangled (up to 9 mm thick), covered with small whitish protruding fibrils at the stipe apex, longitudinally fibrillose downwards the stipe; base with distinctly whitish tomentose hyphae, surface white (3A1) to pale yellowish white (3A2), at times showing indistinct vinaceous-tinged (5B5) longitudinal stripes or becoming vinaceous (5B5) when bruised. Context solid, fleshy in pileus, 1–1.5 mm thick at mid-radius, up to 4–5 mm thick under the umbo, white (4A1) at first, becoming brownish white (5B6) to pale pinkish (6B3) when old or cut, fleshy in stipe, fibrillose, shiny, striate, beige (4A2) to dirty white (4B2), pale yellowish brown (5A2) near the cuticle. Odor indistinct.

Basidiospores [200/10/4] (7.6) 8.0–8.5–9.2 (10.2) × 5.0–5.7–6.2 (6.7) μm, Q = (1.24) 1.34–1.67 (1.92), Q_m_ ± SD = 1.50 ± 0.011, mostly ellipsoid to ovoid, sometimes sub-phaseoliform, occasionally obovoid, with indistinct small apiculus, often yellowish green, yellowish brown in mass, thick-walled, usually with yellowish green to yellowish brown ellipsoid to spherical or amorphous large oily inclusions. Basidia 20–28 × 6–9 μm, clavate, often obtuse at apex, slightly tapered toward the base, mostly hyaline, yellowish green to golden yellow, thin-walled, 4-spored, sterigmata 2–3 (7) μm, with pale yellowish oily droplets, sometimes with golden yellow oily inclusions. Pleurocystidia none. Lamella edge sterile. Cheilocystidia 36–46–59 × 7–10–13 μm (*n* = 20), mostly clavate to enlongate clavate, sometimes broadly clavate, rarely lageniform, often obtuse at apex, becoming slightly narrow to the base, walls sometimes wavy, thin-walled, rarely walls up to 1 μm thick at the middle or at apex, mostly colorless, sometimes full of golden yellow oily contents, occasionally with indistinctly resinous substance. Hymenophoral trama 88–103 μm thick, regularly arranged, mass of hyaline to pale yellowish, composed of hyaline, thin-walled, smooth, cylindric, and inflated hyphae, 16–20 μm wide. Veilpellis hyphae smooth, sometimes slightly encrusted, mostly hyaline to pale yellow, sometimes full of faint yellowish brown oily contents, slenderly clavate to slimly cylindric hyphae 3–9 μm wide. Pileipellis a cutis, regular to sub-regular, golden yellow to yellowish brown in mass, composed of slightly encrusted, colorless to pale yellow, slenderly cylindric hyphae 4–9 μm wide. Pileal trama regularly arranged, hyaline, composed of smooth, inflated, and cylindric hyphae 11–18 μm wide. Stipitipellis a cutis, regular to sub-regular, yellowish brown in mass, composed of slightly encrusted, slenderly cylindric hyphae 5–11 μm wide, often disrupted with extended terminal hyphae 7–11 μm wide, mostly hyaline, smooth to indistinctly encrusted, slenderly clavate, thin-walled, similar with veilipellis. Stipe trama regularly arranged, colorless to pale yellow in mass, composed of hyaline, smooth, thin-walled, slenderly cylindric hyphae 10–20 μm wide. Caulocystidia not observed. Oleiferous hyphae 5–14 μm wide, mostly present in pileus and stipe trama, bent, diverticulate, rarely branched, yellowish brown, often with pale yellowish brown oily inclusions. Clamp connections common on all hyphae.

*Habitat*: gregarious, often as large, discrete clusters, on clay soil, under Fagaceae trees.

*Known distribution*: China (Hainan).

*Additional specimens examined*: China, Hainan Province, Wuzhishan City, Xianlu Lake Resort, 29 July 2022, Y.G. Fan, W.J. Yu, and L.S. Deng, FYG7343 (FCAS3797), Same location, same date, J.H. Hu and L.N. Zhao, FYG7350 (FCAS3798), Same location, same date, Y.L. Zhou, FYG7351 (FCAS3799), Same location, same date, J.L. Gao, FYG7352 (FCAS3800).

### 3.3 UPLC-MS/MS muscarine analysis

**Figure 4:**
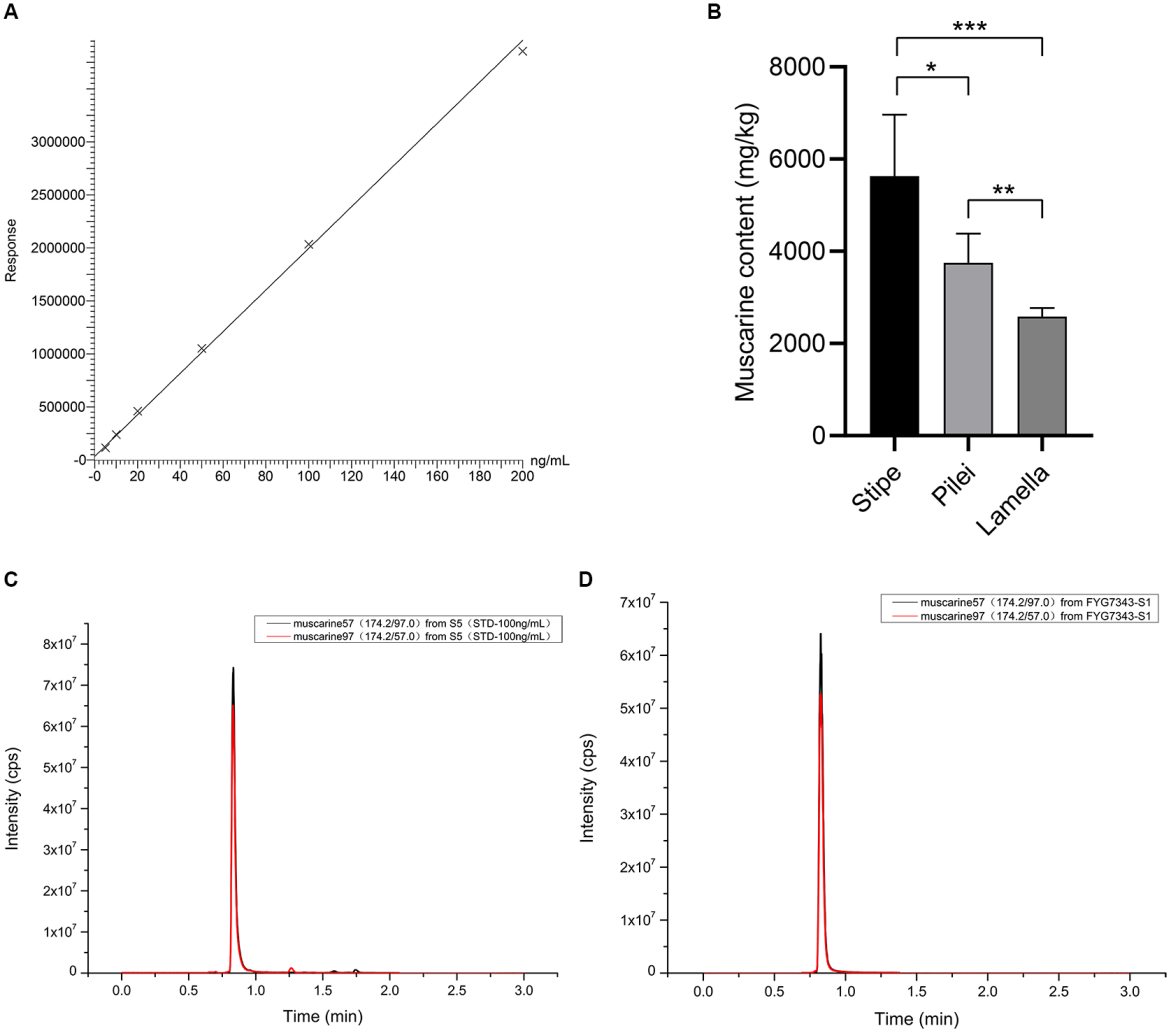
Muscarine in *I. wuzhishanense*. **(A)** The standard curve of muscarine; **(B)** muscatine content in different parts of the fruit body (*n* = 5); *p*-values: ^*^p < 0.1, ^**^p < 0.05, ^***^p < 0.001; **(C)** standard representative chromatograms of muscarine (100 ng/mL); and **(D)** representative chromatograms of muscarine in *I. wuzhishanense* (FYG7343).

Muscarine in the samples was detected by UPLC-MS/MS. As shown in the representative chromatograms in Figures 4C,D, the muscarine retention time (0.83 min) in the samples was identical to that of the muscarine standard (0.83 min). The calibration curve for muscarine generated during the validation was *y* = 19621.4 *x* + 33,174 (Figure 4A). The correlation coefficients (*r* = 0.9995) of linear regression analysis from calibration curves were > 0.99. Linearity was determined for muscarine in the concentration range of 5–200 ng/mL. The matrix effect (ME) was sufficiently weak (|ME| = 0.019%, 0 ≦ |ME| ≦ 20%) to be ignored. All samples were diluted with 500× before UPLC-MS/MS analysis, and double parallel was made for each sample. The precisions and recoveries of muscarine and the muscarine content of the new species are displayed in Tables 1, 2, respectively.

**Table 1:** Precisions, and recoveries for muscarine in the new species measured by UPLC-MS/MS.

The adding standard matter amount (mg/kg)	UPLC-MS/MS			
	Intraday (*n* = 6)		Interday (*n* = 18)	
	Recovery (%)	RSD (%)	Recovery (%)	RSD (%)
5	88.86	1.72	91.33	3.01
10	89.83	0.81	93.27	3.05
20	95.70	1.80	101.20	5.01

**Table 2:** Muscarine content in different parts of different specimens in *I. wuzhishanense* detected by UPLC-MS/MS.

Specimen vouchers	Muscarine content (Mean ± SD, mg/kg)		
	Stipe	Pileus	Lamellae
FYG7659	5,081.47 ± 242.82	2,748.37 ± 106.85	2,512.00 ± 242.82
FYG7343	4,359.79 ± 83.87	4,491.35 ± 467.21	2,775.90 ± 205.62
FYG7350	4,598.49 ± 215.74	3,862.39 ± 15.01	2,301.36 ± 83.52
FYG7351	7,003.19 ± 715.45	3,978.82 ± 187.50	2,563.85 ± 159.69
FYG7352	7,114.03 ± 76.55	3,656.20 ± 3.49	2,744.24 ± 315.95

### 3.4 Element contents

The contents of 24 elements in the new species are summarized in Table 3, provided as means ± standard deviation (mg/kg), as well as the limit of detection (LOD) and the limit of quantification (LOQ). The precisions were performed six times for each element, and the recoveries ranged from 90 to 110%, thereby meeting the specifications of the GB 5009.268–2016 Chinese National Food Safety Standard. The elements detected in the new species were ranked, from highest concentration to lowest, as follows: potassium (K) > magnesium (Mg) > aluminum (Al) > iron (Fe) > zinc (Zn) > copper (Cu) > sodium (Na) > titanium (Ti) > arsenic (As) > calcium (Ca) > manganese (Mn) > barium (Ba) > cadmium (Cd) > chromium (Cr) > vanadium (V) > strontium (Sr) > cobalt (Co) > lead (Pb) > mercury (Hg) > (nickel) Ni > molybdenum (Mo) > thallium (Tl) > boron (B) > tin (Sn). Among all the elements, K had the highest concentration (47,237.30 ± 543.40 mg/kg), Mg was the metal with the second highest concentration (1,298.47 ± 8.48 mg/kg), followed by Al (891.30 ± 1.21 mg/kg), and Sn had the lowest concentration (0.01 ± 0.00 mg/kg). Most importantly, the concentrations of five elements (Cr, As, Cd, Hg, and Pb) exceeded limits specified in the Chinese National Food Safety Standard-Maximum Levels of Contaminants in Foods (GB 2762–2022). Among these five elements, arsenic (36.76 ± 0.43 mg/kg) was the predominant toxic and heavy metal.

**Table 3:** Determined values, precisions, and recoveries measured by ICP-MS/MS.

No.	Element	Determined value (mg/kg), *n* = 3	LOQ (mg/kg), *n* = 11	LOD (mg/kg), *n* = 11	Precisions (%), *n* = 6	Recoveries (%), *n* = 6
1	B	0.02 ± 0.01	0.318	0.095	1.35	98.73
2	Na	67.16 ± 1.21	0.005	0.002	1.41	108.77
3	Mg	1,298.47 ± 8.48	0.000	0.000	1.97	102.42
4	Al	891.30 ± 1.21	0.000	0.000	2.02	102.42
5	K	47,237.30 ± 543.40	0.008	0.002	2.90	105.34
6	Ca	34.28 ± 1.21	0.008	0.002	2.66	100.56
7	Ti	41.04 ± 0.76	0.130	0.039	2.87	98.08
8	V	1.11 ± 0.01	0.003	0.001	2.32	101.49
9	Cr	1.57 ± 0.01	0.045	0.014	1.55	97.89
10	Mn	12.09 ± 0.14	0.013	0.004	3.54	100.15
11	Fe	541.49 ± 2.10	0.003	0.001	3.61	99.06
12	Co	0.55 ± 0.01	0.000	0.000	4.88	91.80
13	Ni	0.12 ± 0.01	0.080	0.024	5.32	103.46
14	Cu	69.76 ± 1.44	0.048	0.014	5.14	104.31
15	Zn	148.46 ± 2.52	0.123	0.037	5.20	98.73
16	As	36.76 ± 0.43	0.008	0.002	4.62	102.22
17	Sr	0.92 ± 0.02	0.008	0.002	5.70	98.25
18	Mo	0.09 ± 0.00	0.003	0.001	5.36	99.16
19	Cd	2.51 ± 0.07	0.003	0.001	6.05	91.86
20	Sn	0.01 ± 0.00	0.005	0.002	10.11	98.32
21	Ba	3.12 ± 0.09	0.020	0.006	6.04	98.71
22	Hg	0.32 ± 0.01	0.003	0.001	3.97	99.58
23	Tl	0.06 ± 0.00	0.000	0.000	4.13	106.66
24	Pb	0.34 ± 0.01	0.145	0.044	13.51	99.89

## 4 Discussions

### 4.1 Identification of the new species

*Inosperma wuzhishanense* was collected in a tropical montane fagaceous forest in Hainan Province, tropical China. It can be recognized by its scattered to often gregarious habit, reddish-brown pileus with appressed scales and initially covered with grayish white veilipellis when young, rather crowded grayish lamellae, and fibrillose-flocculose stipe in the field. In some specimens, an indistinct vinaceous tinge could be found in the squamulose on the pileus or in the fibrils on the stipe surface. Microscopically, it has smooth and ellipsoid basidiospores and thin-walled, often clavate to cylindrical clavate cheilocystidia; no caulocystidia was observed, but instead of protruding hyphal elements. Currently, it is only known from the type locality.

According to the multi-gene phylogenetic analyses, the new species is sister to *I. akirnum*, a species from tropical India (Kerala State). They have similar characteristics, such as fibrillose-rimose pileus, often with a decurved margin, a slightly bulbous stipe base, rather crowded lamella, ellipsoid basidiospores, often clavate cheilocystidia, and the absence of caulocystidia. However, *I. akirnum* has a light brown pileus and appressed fibrillose stipe without any vinaceous tinge, a marginate bulbose stipe base, smaller basidiospores measuring 7–8.5 × 5–5.5 μm, narrower cheilocystidia, and a different habitat among ginger plants (Latha and Manimohan, 2017). *Inosperma saragum*, another taxa also described from tropical India, shares appressed scaly pileus with a decurved margin and crowded lamellae, but differs by its paler yellowish brown to brownish orange color in pileus, larger phaseoliform basidiospores (7–11 × 5–6 μm, 8.9 ± 1.1 × 5.4 ± 0.6 μm on average), broader cheilocystidia (8–18 μm in width) usually with a large globose capitellum (8–17 μm wide) at apex, and a different ecological habitat near Dipterocarpaceae trees (Latha and Manimohan, 2017).

Recent studies have discovered four new *Inosperma* species from tropical China, namely *I. muscarium* Y.G. Fan, L.S. Deng, W.J. Yu, and N.K. Zeng, *I. hainanense* Y.G. Fan, L.S. Deng, W.J. Yu, and N.K. Zeng, *I. zonativeliferum* and *I. subsphaerosporum* Y.G. Fan, L.S. Deng, W.J. Yu, and L.Y. Liu. Among them, *I. muscarium* and *I. hainanense* have distinct fibrillose-rimose pileus without any scales (Deng et al., 2021a); *I. zonativeliferum* has radially arranged whitish scales from its heavy veil remnants and broadly clavate cheilocystidia (Deng et al., 2022a); *I. subsphaerosporum* shares appressed scaly pileus and clavate cheilocystidia, but it has globose to subglobose basidiospores (Deng et al., 2021b). The discovery of *Inosperma wuzhishanense* enriches the knowledge of species diversity in the Old World tropical clade 2.

### 4.2 Muscarine contents

Species in the Old World tropical clade 2 are frequently reported to contain muscarine and cause numerous muscarine-poisoning events in the tropical regions of Asia, such as the concentrations of 0.58 ± 0.02 to 6.53 ± 1.88 g/kg in *I. zonativeliferum* (Deng et al., 2022a), 16.03 ± 1.23 g/kg in *I. muscarium* and 11.87 ± 3.02 g/kg in *I. hainanense* (Deng et al., 2021a), and 0.27 mg/g to 0.3 mg/g in *I. virosum* (Sailatha et al., 2014; Latha et al., 2020); as well as poisoning *Inosperma* materials: *I. carnosibulbosum* (C.K. Pradeep and Matheny) Matheny and Esteve-Rav. (Chandrasekharan et al., 2020) and 16 clinical *Inosperma* samples (Parnmen et al., 2021). The lethal dose of muscarine for humans has been estimated to range from 40 mg to 495 mg (Pauli and Foot, 2005). In the present study, the muscarine content of *I. wuzhishanense* was determined to be 2,301.36 ± 83.52 to 7,114.03 ± 76.55 mg/kg, and the muscarine contents differed significantly among the stipe, pileus, and lamellae.

Many studies have detected significant differences in the amounts of toxin among different tissues of the mushroom fruit body. For instance, analyses of six different tissues of *Amanita fuliginea* Hongo (gills, pileus, stipe, annulus, volva, and spores) revealed that the highest concentration of total toxins (amatoxins and phallotoxins) was in the gills (14.46 mg/g, dry weight), and the lowest concentration was in the spores (0.25 mg/g, dry weight) (Zhou et al., 2017). The amount of amatoxin was found to be higher in the pileus than in the stipe in *A. phalloides* var. *alba* Costantin and L.M. Dufour, *A. exitialis* Zhu L. Yang and T.H. Li, and *A. phalloides* Secr. (Hu et al., 2012; Kaya et al., 2013; Yilmaz et al., 2015), and *Lepiota brunneoincarnata* Chodat and C. Martín (Yilmaz et al., 2015; Sun et al., 2019). Within the Inocybaceae, in *Pseudosperma arenarium* Y.G. Fan, Fei Xu, Hai J. Li, and Vauras, the muscarine content was found to be approximately five times higher in the pileus than in the stipe (Yan et al., 2022); whereas the muscarine content was found to be approximately three times higher in the stipe than in the pileus *I. zonativeliferum* (Deng et al., 2022a). A similar phenomenon was observed in *I. wuzhishanense*. Our results showed that the muscarine content was much higher in the stipe than in the pileus in the new species. As shown in Figure 4B, the highest muscarine content was in the stipe, followed by the pileus, and the lowest muscarine content was in the lamellae, with significant differences among the three tissues. It is interesting to note that *I. wuzhishanense* and *I. zonativeliferum* have the same pattern of muscarine content in the stipe and the pileus. This pattern differs from most of the poisonous mushroom species studied (see above). This includes *P. arenarium*, a poisonous mushroom also belonging to the Inocybaceae. Future investigations are needed to clarify whether this is a common pattern in the *Inosperma* genus and the underlying mechanism.

### 4.3 Element contents

Wild mushrooms accumulate toxic and heavy metals (Alzand et al., 2019). Non-essential elements such as Cr, Cd, As, Hg, and Pb are not only poisonous to aquatic organisms but also harmful to human health, even at low concentrations (Balali-Mood et al., 2021; Noman et al., 2022). These harmful heavy metals have been detected frequently in Agaricaceae, Amanitaceae, Boletaceae, Russulaceae, Inocybaceae, *etc*. (Bizio et al., 2020; Barea-Sepúlveda et al., 2022; Liu et al., 2022). In the present study, five toxic and heavy metals (Cr, As, Cd, Hg, and Pb) and other elements (B, Na, Mg, Al, K, Ca, Ti, V, Mn, Fe, Co, Ni, Cu, Zn, Sr., Mo, Sn, Ba, and Tl) were detected in *I. wuzhishanense* by ICP-MS. As shown in Table 4, the levels of Cd, As, and Hg in the new species were higher than those specified in the National Food Safety Standard for Maximum Levels of Contaminants in Foods of China (GB 2762–2022). Of these three elements, only As was present at a concentration markedly higher than that specified in the GB 2762–2022 standard and in most edible mushrooms (Dowlati et al., 2021; Yu et al., 2021) and most wild mushrooms (Melgar et al., 2014; Fu et al., 2020; Keskin et al., 2021). However, the As concentration in *I. wuzhishanense* was lower than that reported for some species in the Inocybaceae (Bizio et al., 2020). Exposure to As can result in cardiovascular dysfunction, skin and hair changes, central nervous system injury, gastrointestinal discomfort, and liver damage (Balali-Mood et al., 2021). The concentrations of the metalloid element (B) and other 18 other essential metallic elements (K, Mg, Al, Fe, Zn, Cu, Na, Ti, Ca, Mn, Ba, V, Sr., Co, Ni, Mo, Tl, and Sn) were similar to those reported for other *Inosperma*’s fruit bodies in the literature, with only slight differences (Bizio et al., 2020).

**Table 4:** Heavy metal concentrations (mg/kg) in targeted species, comparison with guideline values and related studies.

Species name	Cr	Cd	As	Hg	Pb	References
*I. wuzhishanense*	1.57 ± 0.01	2.51 ± 0.07	36.76 ± 0.43	0.32 ± 0.01	0.34 ± 0.01	Present study
*I. bongardii*	1.16 ± 1.87	8.68 ± 10.54	132.33 ± 578.06	0.21 ± 0.40	1.27 ± 1.23	Bizio et al. (2020)
*I. calamistratum*	1.02 ± 0.72	26.36 ± 29.75	0.39 ± 0.66	0.24 ± 0.27	1.31 ± 0.80	
*I. cervicolor*	0.82 ± 0.52	3.82 ± 3.01	0.13 ± 0.08	0.08 ± 0.06	1.69 ± 1.56	
*I. adaequatum*	0.64 ± 0.42	6.16 ± 3.90	0.24 ± 0.50	0.11 ± 0.09	0.60 ± 0.53	
*I. cookei*	0.77 ± 0.36	2.65 ± 2.29	1.19 ± 1.65	0.18 ± 0.15	3.49 ± 7.93	
*I. maculatum*	0.82 ± 0.36	2.93 ± 2.80	0.43 ± 0.91	0.08 ± 0.08	4.17 ± 10.66	
*I. erubescens*	0.99 ± 0.72	4.33 ± 2.95	0.09 ± 0.02	0.05 ± 0.03	2.15 ± 2.10	
Edible mushrooms	3.205–6.61 (*N* = 45)	0.5412–2.296 (*N* = 69)	/	/	0.8236–4.1458 (*N* = 63)	Dowlati et al. (2021)
*Lentinula edodes*	/	0.170–3.50 (*n* = 60)	0.081–1.520 (*n* = 39)	0.020–0.079 (*n* = 23)	0.020–0.520 (*n* = 23)	Yu et al. (2021)
Wild mushrooms	/	0.28–7.88 (*n* = 190)	/	/	0.07–5.68 (*n* = 190)	Sarikurkcu et al. (2021)
	0.03–13.17 (*n* = 21)	0.06–2.52 (*n* = 21)	/	/	/	Keskin et al. (2021)
			0.03–1.07 (*n* = 51)			Melgar et al. (2014)
	1.59–75 (*n* = 57)	0.21–20.5 (*n* = 57)	0.16–32.7 (*n* = 57)	0.07–3.59 (*n* = 57)	0.21–3.56 (*n* = 57)	Fu et al. (2020)
National standard of China	/	0.5	0.5	0.1	1.0	GB2762-2022

“N” refers to the number of studies; “n” is the sample number; “/” means no data.

## 5 Conclusion

A new *Inosperma* taxa was found in tropical China and has been named *I. wuzhishanense*. In this new species, the muscarine contents were ranged from 2,301.36 ± 83.52 to 7,114.03 ± 76.55 mg/kg and differed significantly among the stipe, pileus, and lamellae. Additionally, *I. wuzhishanense* demonstrated an exceptional ability to accumulate Cr, Hg, Pb, Cd, and As. Therefore, this species poses risks to human health because of the presence of muscarine and the high concentrations of toxic and heavy metals.

## Data availability statement

The original contributions presented in the study are publicly available. This data can be found here: https://doi.org/10.5281/zenodo.10049385.

## Author contributions

Y-LZ: Data curation, Funding acquisition, Writing – original draft. L-SD: Data curation, Investigation, Methodology, Project administration, Resources, Writing – original draft, Writing – review & editing. S-DY: Data curation, Formal analysis, Methodology, Validation, Writing – review & editing. C-FL: Data curation, Formal analysis, Methodology, Software, Writing – review & editing. Y-GF: Conceptualization, Funding acquisition, Writing – review & editing. W-JY: Conceptualization, Funding acquisition, Investigation, Resources, Supervision, Writing – review & editing.

## Footnotes

^1^ https://submit.ncbi.nlm.nih.gov/about/genbank/

^2^ https://mafft.cbrc.jp/alignment/server/

^3^ http://iqtree.cibiv.univie.ac.at/
