# Supplementary material for: Phylogenetic analysis, morphological studies, element profiling, and muscarine detection reveal a new toxic Inosperma (Inocybaceae, Agaricales) species from tropical China
Source: Front Microbiol. 2023 Dec 7;14:1326253. doi: 10.3389/fmicb.2023.1326253 (PMC10740167; doi:10.3389/fmicb.2023.1326253)
Supplement: Supplementary file 1 [file Data_Sheet_1.PDF]

Supplementary Material

1 Supplementary Figures and Tables

1.1 Supplementary Tables

Supplemental table 1. Taxa, collection number, locality and GenBank number used for molecular phylogenetic analyses

Taxa	Collection number / Herbaium	Locality	GenBank accession number		
			**ITS**	**LSU**	***RPB2***
Auritella hispida	TH10009	Cameroon	KT378203	KT378207	KT378215
Auritella spiculosa	TH9866	Cameroon	KT378204	KT378206	KT378214
Inosperma acutofulvum	MCVE29416	Italy	MG944832	—	—
I. adaequatum	JV16501F	Finland	—	AY380364	AY333771
I. aff. *lanatodiscum*	PBM3051	USA	JQ801401	JN975026	JQ846485
I. aff. *calamistratum*	DED8134	Thailand	GQ892983	GQ892937	—
I. aff. *calamistratum*	REH8420	Costa Rica	JQ801390	JN975018	JQ846471
I. aff. *fastigiellum*	PBM3325	USA	JQ801399	JQ815419	JQ846477
I. aff. *latericium*	TR109-02	PNG	JQ801405	JN975023	JQ846487
I. africanum	HLA0383 (Type)	Benin	MT534298	MT560733	
I. africanum	HLA0353	Benin	MT534299		

I. akirnum	CAL1358	India	KY440085	KY549115	KY553236
I. afromelliolens	PC96013	Zambia	JQ801383	EU600882	EU600883
I. akirnum	CAL1358	India	KY440085	KY549115	KY553236
I. apiosmotum	PBM3020	USA	JQ801385	JN975021	JQ846463
I. bicoloratum	ZT12187	Malaysia	GQ892984	GQ892938	JQ846464
I. bongardii	JV7450F	Finland	—	EU555448	—
I. bulbomarginatum	MR00357	Benin	MN096190	MN200775	MN097882
I. bulbomarginatum	PC96082	Benin	JQ801412	JN975027	—
I. calamistratoides	PBM3384	Australia	JQ801393	JQ815415	KJ729949
I. calamistratum	PBM1105	USA	JQ801386	JQ815409	JQ846466
I. calamistratum	EL1904	Sweden	AM882938	AM882938	—
I. calamistratum	PBM2351	USA	—	AY380368	AY333764
I. calamistratum	JV11950	Latvia	—	EU555452	AY333763
I. calamistratum	TR74-06	PNG	JQ801391	JN975020	JQ846472
I. carnosibulbosum	TBGT12047	India	KT329448	KT329443	KT329454

I. cervicolor	TURA4761	Finland	JQ801395	JQ815417	JQ846474
I. cf. *cervicolor*	TENN: 065721	Australia	—	JQ815429	—
I. cf. *gregarium*	D82	Thailand	MW538596	MW512894	MW538609
I. cf. *gregarium*	D84	Thailand	MW538598	MW512895	MW538610
I. cf. *gregarium*	D91	Thailand	MW538602	MW512896	MW538611
I. cf. *gregarium*	D92	Thailand	MW538603	MW512897	MW538612
I. cf. *lanatodiscum*	TURA1812	Finland	JQ408763	JQ319694	JQ846484
I. cf. *reisneri*	MCA646	Japan	—	EU555463	—
I. changbaiense	FYG2010156 (Type)	China	MH047251	MG844976	MT086755
I. cyanotrichium	I37	Australia	JQ801396	JN975033	JQ846476
I. dodonae	STU: SMNS-STU-F-0901253	Netherlands	MW647615	—	—
I. erubescens	JV9070F	Finland	—	EU569846	—
I. flavobrunneum	HLA0367	Benin	MN096199	MT536754	—
I. geraniodorum	EL10606	Sweden	FN550945	FN550945	—

I. gregarium	CAL1309	India	KX852305	KX852307	KX852306
I. gregarium	ZT8944	India	—	EU600903	EU600902
I. hainanense	Zeng4737 (Type)	China	MZ373980	—	MZ388091
I. ismeneanum	STU: SMNS-STU-F-0901561	Germany	MW647625	—	—
I. lanatodiscum	PBM2451	USA	JQ408759	JQ319690	JQ846483
I. latericium	PDD92382	New Zealand	GU233367	GU233413	—
I. longisporum	MHNNU32337 (Type)	China	OP135509	OP135495	OP161560
I. maculatum	EL12604	Sweden	AM882964	AM882964	—
I. maculatum	PBM2446	USA	DQ241778	AY745700	EU569863
I. maximum	PBM2222	USA	—	EU569854	—
I. misakaense	PC96234	Zambia	JQ801409	EU569875	AY333767
I. monastichum	STU: SMNS-STU-F-0901533	Germany	MW647631	—	—
I. mucidiolens	DG1824 (Type)	Canada	HQ201339	HQ201340	—
I. muscarium	FYG6091 (Type)	China	MZ373982	MZ388093	MZ373991
I. mutatum	PBM2542	USA	—	AY732212	DQ472729

I. neobrunnescens	PBM2452	USA	—	EU569868	EU569867
I. neobrunnescens var. *leucothelotum*	SAT0427406	USA	JQ801411	JN975025	JQ846489
I. proximum	ZT13015	Thailand	EU600839	EU600840	—
I. quietiodor	EL11504	Sweden	AM882960	AM882960	—
I. rhodiolum	EL223-06	France	FJ904175	FJ904175	—
I. rimosoides	PBM2459	USA	DQ404391	AY702014	DQ385884
I. rubricosum	PBM3784	Australia	KP308817	KP170990	KM406230
I. saragum	CAL1360	India	KY440103	KY553249	KY549133
I. shawarense	ASSE79	Pakistan	KY616964	KY616966	—
Inosperma sp.	L-GN3a	PNG	JX316732	—	JX316732
Inosperma sp.	TJB10045	Thailand	KT600658	KT600660	KT600659
Inosperma sp.	TR220-06	PNG	JQ801416	JQ846496	JN975017
Inosperma sp.	D67	Thailand	MW538582	MW512898	MW538613
Inosperma sp.	D68	Thailand	MW538583	MW512899	MW538614

Inosperma sp.	D76	Thailand	MW538590	MW512900	MW538615
Inosperma sp.	D77	Thailand	MW538591	MW512901	MW538616
Inosperma sp.	D78	Thailand	MW538592	MW512902	MW538617
Inosperma sp.	D80	Thailand	MW538594	MW512903	MW538618
Inosperma sp.	D83	Thailand	MW538597	MW512904	MW538619
Inosperma sp.	D85	Thailand	MW538599	MW512905	MW538620
Inosperma sp.	D86	Thailand	MW538600	MW512906	MW538621
Inosperma sp.	D89	Thailand	MW538601	MW512907	MW538622
Inosperma sp.	D152	Thailand	MW538604	MW512908	MW538623
Inosperma sp.	D324	Thailand	MW538607	MW512909	MW538624
Inosperma sp.	PBM2871	USA	HQ201348	HQ201348	JQ846475
Inosperma sp.	BB3233	Zambia	JQ801415	EU600885	—
I. sphaerobulbosum	MHNNU32266 (Type)	China	OP135501	OP134001	OP161559
I. squamulosobrunneum	MHNNU32359 (Type)	China	OP135499	OP134000	OP161562
I. squamulosohinnuleum	MHNNU32195 (Type)	China	OP135500	OP134002	OP161558

I. subhirsutum	PC96073	Zambia	JQ801417	EU600870	EU600869
I. subsphaerosproum	FYG5848 (Type)	China	MW403825	MW404237	MW397171
I. vinaceobrunneum	PBM2951	USA	—	HQ201353	JQ846478
I. vinaceum	AMB18747	Italy	MW561108	MW561120	—
I. viridipes	I153	Australia	KP641646	KP171095	KM656139
I. virosum	TBGT753	India	KT329452	KT329446	KT329458
I. virosum	CAL1383	India	KY440108	KY553253	KY549138
I. wuzhishanense	**FYG7659 (Type)**	**China**	**OR436481**	**OR436487**	—
I. wuzhishanense	**FYG7343**	**China**	**OR436482**	**OR436488**	**OR451201**
I. wuzhishanense	**FYG7350**	**China**	**OR436483**	**OR436489**	**OR451202**
I. wuzhishanense	**FYG7351**	**China**	**OR436484**	**OR436490**	**OR451203**
I. wuzhishanense	**FYG7352**	**China**	**OR436485**	**OR436491**	**OR451204**
I. zonativeliferum	FYG6441 (Type)	China	OL850878	OM845772	ON075044

PNG: Papua New Guinea; "—": null

1.2 Supplementary Figures

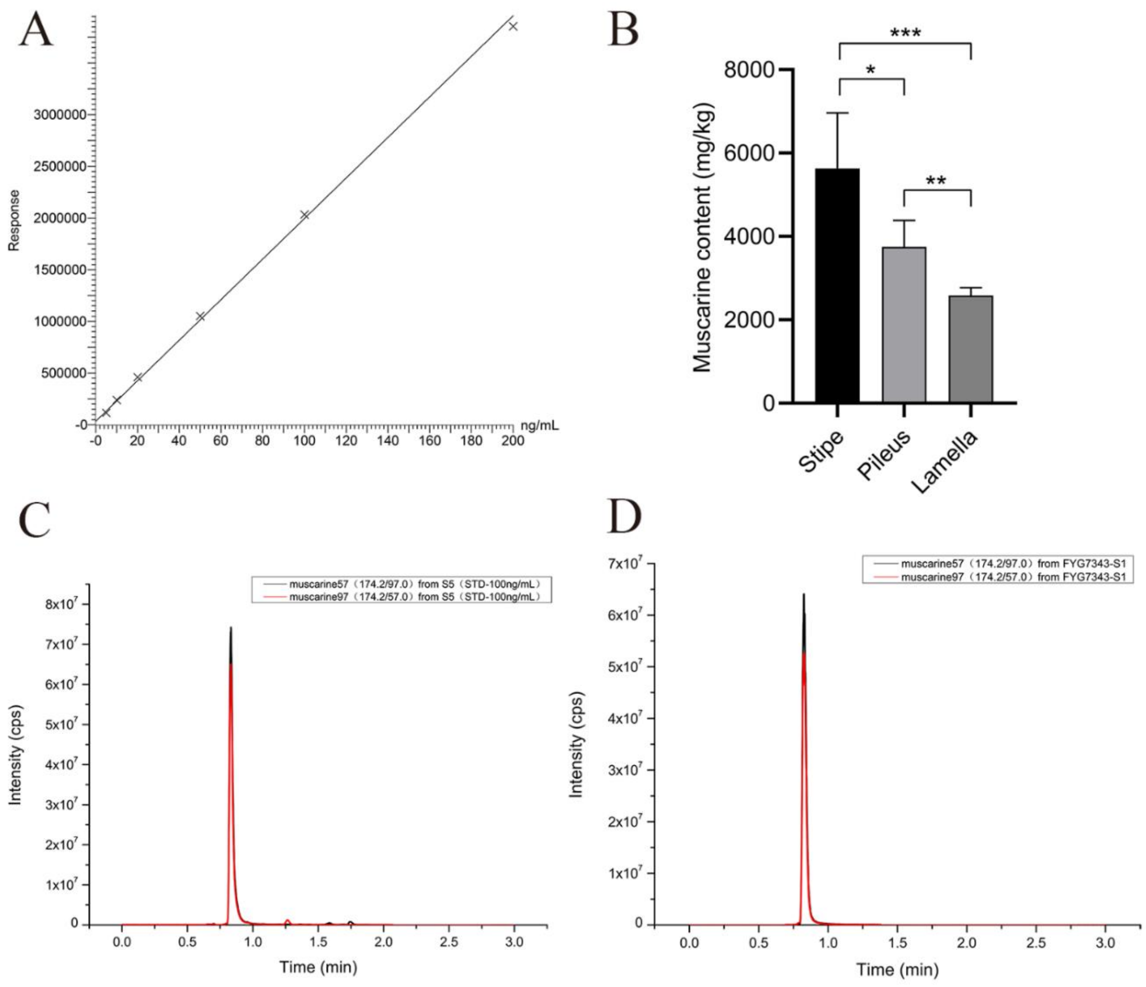

Supplementary Figure 1. Muscarine in *I. wuzhishanense*. (A) The standard curve of muscarine; (B) Muscatine content in different parts of the fruitbody (n=5); p -values: * $p < 0.1$, ** $p < 0.05$, *** $p < 0.001$; (C) Standard representative chromatograms of muscarine (100 ng/mL); (D) The representative chromatograms of muscarine in *I. wuzhishanense* (FYG7343)

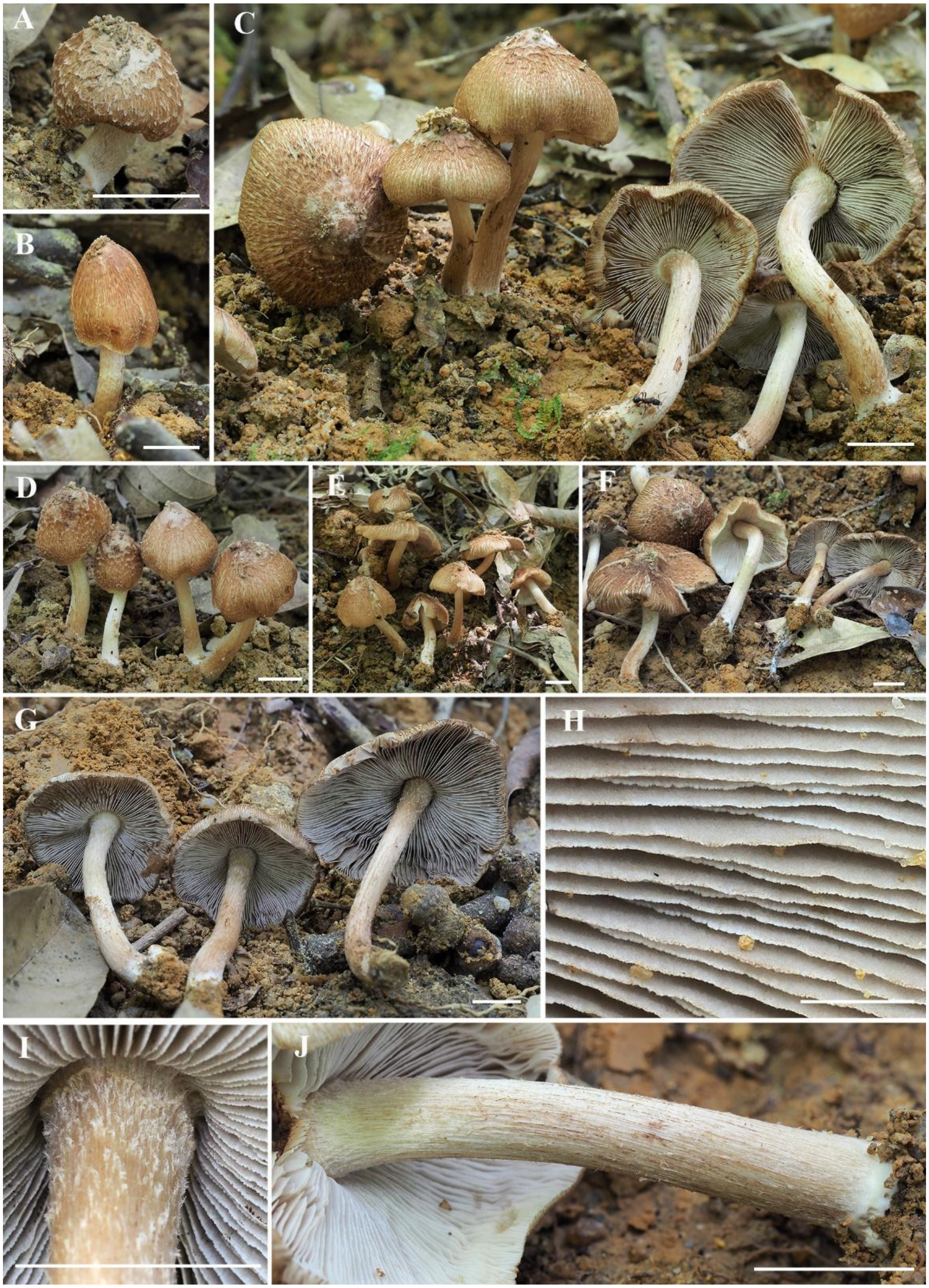

Supplementary Figure 2. Macroscopic characteristics of *I. wuzhishanense*. (A–F) Basidiomata; (G) Lamellae; (H) Lamellae edge; (I–J) Stipe surface. Scale bars: A–J=10 mm. Photos by Y.-G. Fan

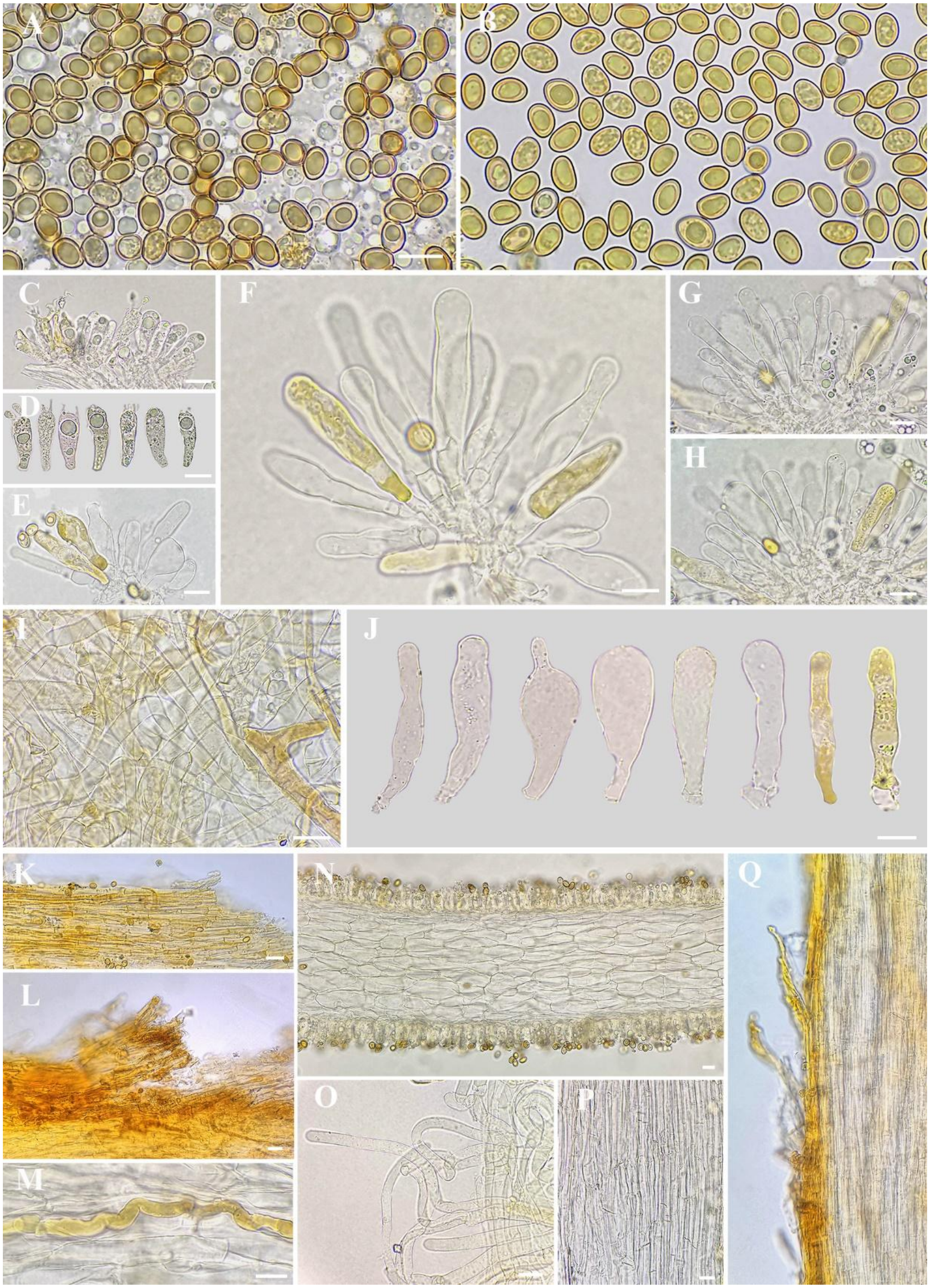

Supplementary Figure 3. Microscopic features of *I.wuzhishanense*. (A–B) Basidiospores; (C–D) Basidia; (E–H, J) Cheilocystidia in clusters; (I) Veilpellis; (K–L) Pileipellis; (M) Oleiferous hyphae; (N) Hymenophoral trama; (O) Extended terminal inflated hyphae in upper stipe surface; (P) Stipe trama; (Q) Stipitipellis. Scale bars: A–Q=10 μm. Photos by L.-S. Deng

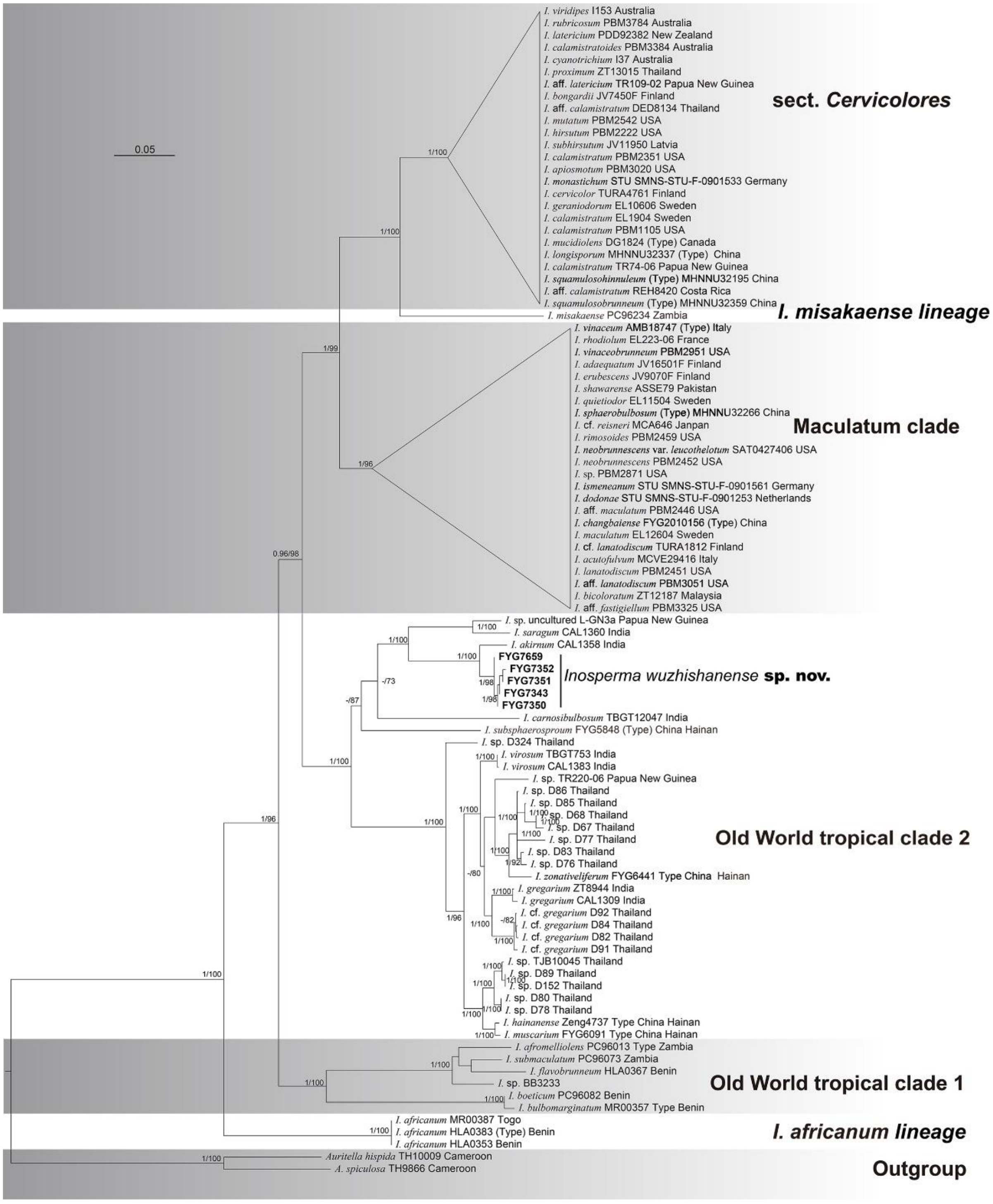

Supplementary Figure 4. Phylogram generated by Bayesian (BI) analyses based on sequences of a combined dataset from nuclear genes, rooted with *Auritella hispida* and *A. spiculosa*. Bayesian inference (BI-PP) ⩾ 0.95 and ML bootstrap proportions (ML-BP) ⩾ 70 are represented as BI-PP/ML-BP. *I. wuzhishanense* is the newly described taxa
